# Crystal structure of 2,4-di­amino-6-oxo-3,6-di­hydro­pyrimidin-1-ium *p*-toluene­sulfonate

**DOI:** 10.1107/S2056989015006787

**Published:** 2015-04-11

**Authors:** Krishnasamy Mamallan, Sadasivam Sharmila Tagore, Sundaramoorthy Gomathi, Velusamy Sethuraman

**Affiliations:** a Department of Chemistry, Periyar Maniammai University, Thanjavur 613 403, Tamil Nadu, India

**Keywords:** crystal structure, di­amino­pyrimidines, tri­amino­pyrimidines, *p*-toluene­sulfonate, hydrogen bonding, N—H⋯O hydrogen bonds, π–π stacking inter­actions

## Abstract

In the title salt, C_4_H_7_N_4_O^+^·C_7_H_7_O_3_S^−^, the 2,6-di­amino-4-oxo-1,3-di­hydro­pyrimidin-1-ium cation inter­acts with the sulfonate group of the *p*-toluene­sulfonate anion *via* a pair of N—H⋯O hydrogen bonds, forming a hetero-synthon *R*_2_^2^(8) that mimics the role of a carboxyl­ate. The self-assembled cations form a homo-synthon *R*_2_^1^(6) motif which is further linked with the sulfonate anion *via* N—H⋯O hydrogen bonds to generate an *R*_3_^2^(10) ring motif. The three motifs are fused together and extended as supra­molecular ribbons along the *b*-axis direction. Adjacent ribbons are further linked *via* N—H⋯O hydrogen bonds to form an annulus, with an *R*_4_^4^(20) ring motif, resulting in a tunnel-like arrangement propagating along [010]. There are slipped parallel π–π stacking inter­actions [inter-centroid distance = 3.6539 (7) Å], between the tunnel-like polymer chains, forming slabs parallel to (100).

## Abstract 2

In the crystal of the title salt, the 2,6-di­amino-4-oxo-1,3-di­hydro­pyrimidin-1-ium cations and the *p*-toluene­sulfonate anions are linked by a series of N—H⋯O hydrogen bonds, forming tunnel-like polymer chains propagating along [010].

## Chemical context   

Di- and tri-amino­pyrimidines show various biological and pharmacological properties like tyrosine kinase (Thomas, 1995*a* ▸,*b* ▸), di­hydro­folate reductase inhibitors (Ayer, 1991 ▸) and are used as anti­viral and anti­protozoan agents. 2,6-Di­amino-4-hy­droxy pyrimidine (DAHP), an inhibitor of guanosine triphosphate cyclo­hydro­lase I, blocks the synthesis of tetra­hydro­biopterin which is a known cofactor of inducible nitric oxide synthesis (iNOS) (Bogdan *et al.*, 1995 ▸). The study of hydrogen-bonding patterns involving sulfonate groups in biological systems and metal complexes are of current inter­est (Gomathi & Mu­thiah, 2011 ▸; Wang, 2006 ▸). The present report deals with the supra­molecular inter­actions exhibited by the title salt.
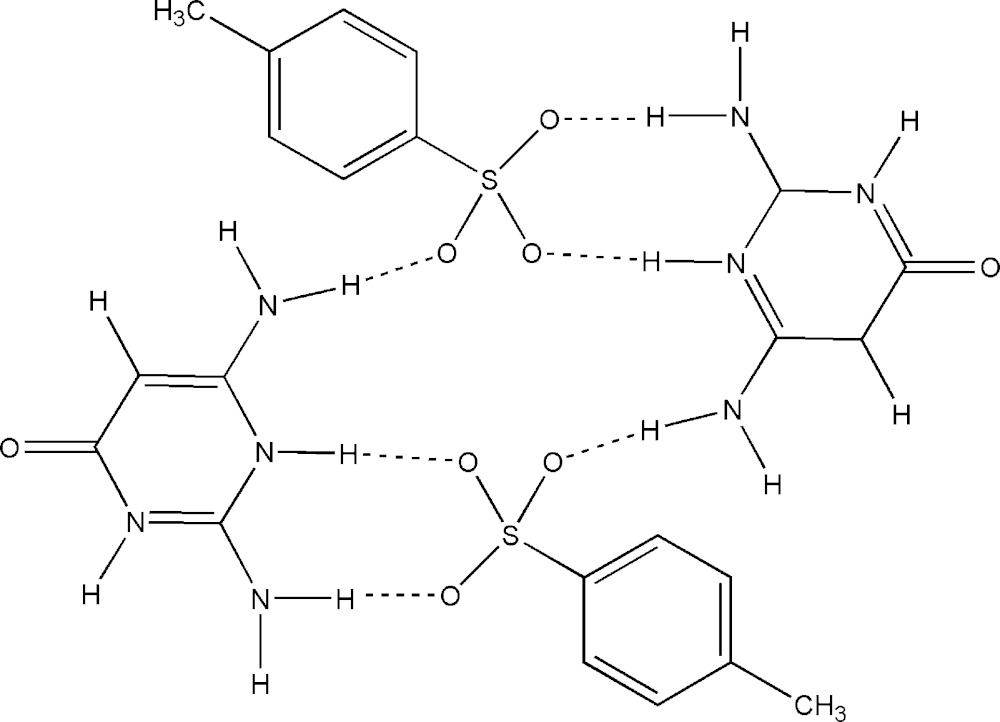


## Structural commentary   

**Figure 1:**
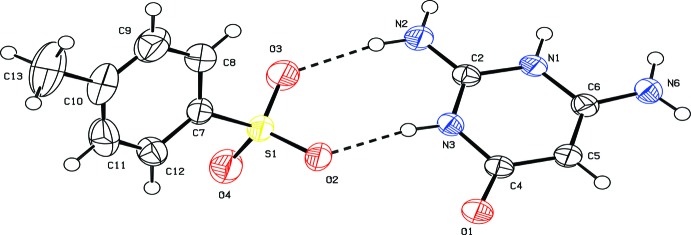
A view of the mol­ecular structure of the title mol­ecular salt, with atom labelling. Displacement ellipsoids are drawn at the 50% probability level.

The asymmetric unit of the title salt contains one 2,6-di­amino-4-oxo-1,3-di­hydro­pyrimidin-1-ium cation and one *p*-toluene sulfonate anion (Fig. 1 ▸). The cation is protonated at the N3 position, which is reflected by the slight increase in the C2—N3—C4 bond angle to 123.2 (1)°. The dihedral angle between the cation and anion ring mean planes is 54.04 (6)°.

The three C—S—O angles, C7—S1—O3 [106.83 (7)°], C7—S1—O2 [105.89 (7)°] and C7—S1—O4 [106.91 (7)°], and the O—S—O angles, O3—S1—O2 [110.84 (7)°], O2—S1—O4 [111.93 (7)°] and O3—S1—O4 [113.91 (8)°], indicate that the geometry of the sulfonate group is slightly distorted from an ideal tetra­hedral geometry.

## Supra­molecular features   

**Figure 2:**
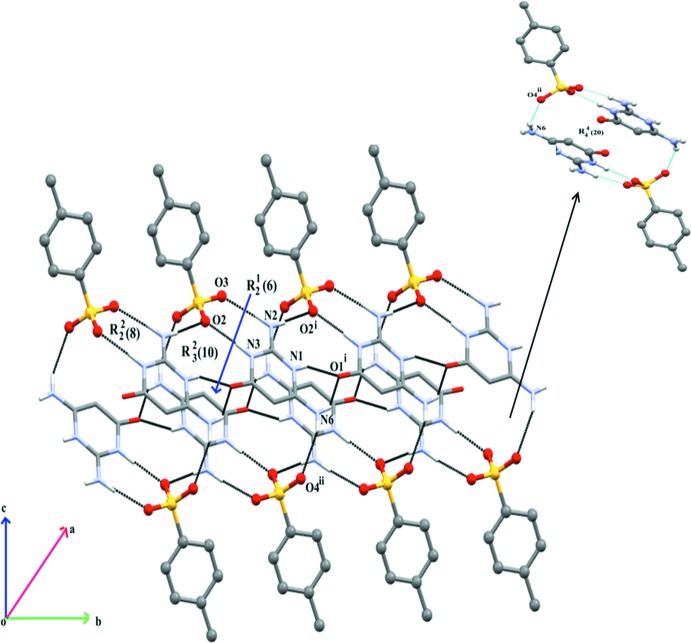
A view of the supra­molecular tunnel-like architecture built by N—H⋯O hydrogen bonds [dashed lines; see Table 1 ▸ for details; symmetry codes: (i) *x*, *y* + 1, *z*; (ii) −*x* + 1, −*y* + 1, −*z* + 1], in the crystal structure of the title mol­ecular salt.

The primary inter­action between the cation and anion takes place *via* a pair of N—H⋯O hydrogen bonds, forming a robust six-membered hetero-synthon, 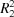
(8), and here the sulfonate group mimics the role of a carboxyl­ate. This motif links the protonated ring N atom, N3, and the 2-amino N atom, N2, of the cation with the sulfonate atoms O2 and O3 of the anion. Adjacent 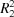
(8) ring motifs are connected *via* an N—H⋯O hydrogen bond by linking the 2-amino N atom, N2 with atom O3^i^ [symmetry code: *x*, *y* + 1, *z*]. The cation undergoes self-association *via* a pair of bifurcated N—H⋯(O,O) hydrogen bonds, forming a homo-synthon, 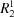
(6). This motif involves ring N1 and the 6-amino N atoms and carbonyl atom O1^i^ of the cation (Table 1 ▸). The self-assembled cations extend as a supra­molecular chain propagating along [010]. The homo- and hetero-synthons [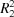
(8) and 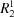
(6)] are linked by an 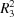
(10) ring motif. The three motifs are fused together continuously, forming supra­molecular ribbons along [010]. Two such ribbons in adjacent planes are connected *via* N—H⋯O hydrogen bonds by linking the 6-amino N of the cation and the sulfonate atom O4^ii^ [symmetry code: −*x* + 1, −*y* + 1, −*z* + 1] of the anion, generating an annulus (Su *et al.*, 2007 ▸) with an 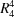
(20) graph-set motif (Fig. 2 ▸). This motif extends in the direction of the supra­molecular ribbons and generates a tunnel-like architecture along the *b-*axis direction (Figs. 2 ▸ and 3 ▸).

**Figure 3:**
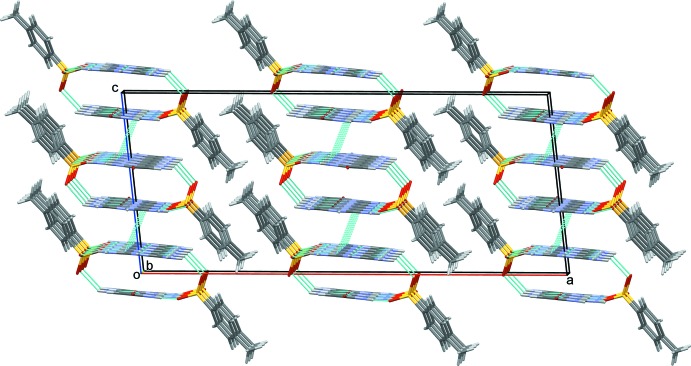
A view along the *b* axis of the crystal packing of the title mol­ecular salt. Hydrogen bonds (see Table 1 ▸ for details) and π–π inter­actions are shown as dashed lines.

Adjacent tunnels inter­act by off-set aromatic π–π stacking inter­actions which are observed between symmetry-related pyrimidine rings of the cations with a centroid–centroid distance *Cg*⋯*Cg* ^iii^ of 3.6539 (7) Å [*Cg* is the centroid of ring N1/C2/N3/C4–C6; the dihedral angle between the ring planes = 1.86 (6)°; perpendicular separation = 3.2501 (5) Å; symmetry code: (iii) −*x* + 1, *y*, −*z* + 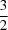]. These inter­actions result in the formation of slabs parallel to (100); as shown in Fig. 3 ▸.

## Database survey   

A search of the Cambridge Structural Database (Version 5.36; Groom & Allen, 2014 ▸) revealed the presence of over 700 compounds involving *p*-toluene sulfonate but only three hits for the 2,6-di­amino-4-oxo-1,3-di­hydro­pyrimidin-1-ium cation. These include the sulfate monohydrate (ACEYUD; Mu­thiah *et al.*, 2004 ▸), the di(methane­sulfan­yl)amide (ESAQOE; Wijaya *et al.*, 2004 ▸) and the chloride dihydrate (SUZFOJ; Suleiman Gwaram *et al.*, 2010 ▸). In ACEYUD the cation is protonated at the N atom adjacent to the carbonyl group, as in the title compound, while in compounds ESAQOE and SUZFOJ it is the N atom *para* to the carbonyl group that is protonated. Otherwise, the bond distances in these three compounds are very similar and close to those observed for the title compound.

## Synthesis and crystallization   

A hot methano­lic solution (20 ml) of 2,6-di­amino-4-hy­droxy pyrimidine (31.5 mg, Aldrich) and *p*-toluene sulfonic acid (43 mg, Loba chemie) was warmed at 323 K for 30 min over a water bath. The mixture was cooled slowly and kept at room temperature and after three weeks light-yellow needle-shaped crystals were obtained.

## Refinement   

Crystal data, data collection and structure refinement details are summarized in Table 2 ▸. All H atoms were positioned geometrically and refined using a riding model: N—H = 0.86 Å, C—H = 0.93–0.96 Å with *U*_iso_(H) = 1.5*U*_eq_(C) for methyl H atoms and 1.2*U*_eq_(N,C) for other H atoms.

## Supplementary Material

Crystal structure: contains datablock(s) global, I. DOI: 10.1107/S2056989015006787/su5107sup1.cif

Structure factors: contains datablock(s) I. DOI: 10.1107/S2056989015006787/su5107Isup2.hkl

Supporting information file. DOI: 10.1107/S2056989015006787/su5107Isup3.cml

CCDC reference: 1057933

Additional supporting information:  crystallographic information; 3D view; checkCIF report

## Figures and Tables

**Table 1:** Hydrogen-bond geometry (, )

*D*H*A*	*D*H	H*A*	*D* *A*	*D*H*A*
N1H1O1^i^	0.86	1.86	2.6515(14)	152
N2H2*A*O3	0.86	1.95	2.7935(17)	166
N2H2*B*O2^i^	0.86	2.01	2.8669(16)	175
N3H3O2	0.86	1.92	2.7689(14)	169
N6H6*A*O4^ii^	0.86	2.25	2.9498(18)	139
N6H6*B*O1^i^	0.86	2.08	2.8201(15)	143

Symmetry codes: (i) 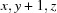; (ii) 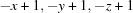.

**Table 2:** Experimental details

Crystal data	
Chemical formula	C_4_H_7_N_4_O^+^C_7_H_7_O_3_S
*M*_r_	298.33
Crystal system, space group	Monoclinic, *C*2/*c*
Temperature (K)	296
*a*, *b*, *c* ()	30.8628(7), 6.5559(2), 13.1565(3)
()	96.428(1)
*V* (^3^)	2645.27(12)
*Z*	8
Radiation type	Mo *K*
(mm^1^)	0.27
Crystal size (mm)	0.30 0.20 0.20

Data collection	
Diffractometer	Bruker Kappa APEXII CCD
Absorption correction	Multi-scan (*SADABS*; Bruker, 2004 ▸)
*T*_min_, *T*_max_	0.925, 0.949
No. of measured, independent and observed [*I* > 2(*I*)] reflections	14972, 3580, 2986
*R*_int_	0.022
(sin /)_max_ (^1^)	0.687

Refinement	
*R*[*F* ^2^ > 2(*F* ^2^)], *wR*(*F* ^2^), *S*	0.037, 0.109, 1.06
No. of reflections	3580
No. of parameters	182
H-atom treatment	H-atom parameters constrained
_max_, _min_ (e ^3^)	0.31, 0.25

Computer programs: *APEX2* and *SAINT* (Bruker, 2004 ▸), *SHELXS97* and *SHELXL97* (Sheldrick, 2008 ▸), *PLATON* (Spek, 2009 ▸), *Mercury* (Macrae *et al.*, 2008 ▸) and *publCIF* (Westrip, 2010 ▸).

## supplementary crystallographic information

### Crystal data

**Table:**

C_4_H_7_N_4_O^+^·C_7_H_7_O_3_S^−^	*F*(000) = 1248
*M**_r_* = 298.33	*D*_x_ = 1.498 Mg m^−^^3^
Monoclinic, *C*2/*c*	Mo *K*α radiation, λ = 0.71073 Å
Hall symbol: -C 2yc	Cell parameters from 3580 reflections
*a* = 30.8628 (7) Å	θ = 2.7–29.3°
*b* = 6.5559 (2) Å	µ = 0.27 mm^−^^1^
*c* = 13.1565 (3) Å	*T* = 296 K
β = 96.428 (1)°	Prism, colourless
*V* = 2645.27 (12) Å^3^	0.30 × 0.20 × 0.20 mm
*Z* = 8	

### Data collection

**Table:**

Bruker Kappa APEXII CCD diffractometer	3580 independent reflections
Radiation source: fine-focus sealed tube	2986 reflections with *I* > 2σ(*I*)
Graphite monochromator	*R*_int_ = 0.022
ω and φ scan	θ_max_ = 29.2°, θ_min_ = 2.7°
Absorption correction: multi-scan (*SADABS*; Bruker, 2004)	*h* = −40→42
*T*_min_ = 0.925, *T*_max_ = 0.949	*k* = −9→7
14972 measured reflections	*l* = −18→16

### Refinement

**Table:**

Refinement on *F*^2^	Primary atom site location: structure-invariant direct methods
Least-squares matrix: full	Secondary atom site location: difference Fourier map
*R*[*F*^2^ > 2σ(*F*^2^)] = 0.037	Hydrogen site location: inferred from neighbouring sites
*wR*(*F*^2^) = 0.109	H-atom parameters constrained
*S* = 1.06	*w* = 1/[σ^2^(*F*_o_^2^) + (0.0552*P*)^2^ + 1.4509*P*] where *P* = (*F*_o_^2^ + 2*F*_c_^2^)/3
3580 reflections	(Δ/σ)_max_ = 0.001
182 parameters	Δρ_max_ = 0.31 e Å^−^^3^
0 restraints	Δρ_min_ = −0.25 e Å^−^^3^

### Special details

**Table:**

Geometry. Bond distances, angles *etc*. have been calculated using the rounded fractional coordinates. All su's are estimated from the variances of the (full) variance-covariance matrix. The cell e.s.d.'s are taken into account in the estimation of distances, angles and torsion angles
Refinement. Refinement on *F*^2^ for ALL reflections except those flagged by the user for potential systematic errors. Weighted *R*-factors *wR* and all goodnesses of fit *S* are based on *F*^2^, conventional *R*-factors *R* are based on *F*, with *F* set to zero for negative *F*^2^. The observed criterion of *F*^2^ > σ(*F*^2^) is used only for calculating -*R*-factor-obs *etc*. and is not relevant to the choice of reflections for refinement. *R*-factors based on *F*^2^ are statistically about twice as large as those based on *F*, and *R*-factors based on ALL data will be even larger.

### Fractional atomic coordinates and isotropic or equivalent isotropic displacement parameters (Å^2^)

**Table:**

	*x*	*y*	*z*	*U*_iso_*/*U*_eq_	
S1	0.37258 (1)	0.33503 (6)	0.60709 (3)	0.0395 (1)	
O2	0.41787 (3)	0.26937 (17)	0.63677 (10)	0.0469 (4)	
O3	0.36871 (4)	0.55423 (17)	0.61453 (11)	0.0559 (4)	
O4	0.35526 (4)	0.2530 (2)	0.50899 (10)	0.0594 (4)	
C7	0.34167 (4)	0.2292 (2)	0.69886 (12)	0.0379 (4)	
C8	0.33328 (5)	0.3414 (3)	0.78333 (14)	0.0508 (5)	
C9	0.30882 (6)	0.2558 (4)	0.85489 (15)	0.0631 (7)	
C10	0.29210 (5)	0.0608 (4)	0.84233 (15)	0.0611 (7)	
C11	0.30171 (6)	−0.0499 (3)	0.75883 (16)	0.0595 (6)	
C12	0.32634 (5)	0.0315 (3)	0.68668 (14)	0.0477 (5)	
C13	0.26346 (7)	−0.0295 (5)	0.9167 (2)	0.0946 (12)	
O1	0.52384 (3)	0.28686 (15)	0.61202 (10)	0.0461 (4)	
N1	0.50945 (3)	0.88937 (16)	0.62432 (8)	0.0306 (3)	
N2	0.43613 (4)	0.84071 (18)	0.63472 (10)	0.0382 (3)	
N3	0.48173 (3)	0.56345 (16)	0.62787 (9)	0.0314 (3)	
N6	0.58157 (4)	0.95420 (18)	0.60830 (11)	0.0424 (4)	
C2	0.47500 (4)	0.76549 (19)	0.62867 (9)	0.0289 (3)	
C4	0.52230 (4)	0.4758 (2)	0.61702 (10)	0.0321 (3)	
C5	0.55713 (4)	0.6088 (2)	0.61103 (11)	0.0343 (4)	
C6	0.55075 (4)	0.8158 (2)	0.61406 (10)	0.0308 (3)	
H8	0.34390	0.47360	0.79230	0.0610*	
H9	0.30360	0.33110	0.91220	0.0760*	
H11	0.29140	−0.18290	0.75060	0.0710*	
H12	0.33250	−0.04600	0.63080	0.0570*	
H13A	0.23360	0.00590	0.89590	0.1420*	
H13B	0.27210	0.02330	0.98400	0.1420*	
H13C	0.26650	−0.17530	0.91740	0.1420*	
H1	0.50570	1.01900	0.62810	0.0370*	
H2A	0.41430	0.76020	0.63830	0.0460*	
H2B	0.43230	0.97070	0.63510	0.0460*	
H3	0.46010	0.48390	0.63430	0.0380*	
H5	0.58480	0.55720	0.60500	0.0410*	
H6A	0.60780	0.91650	0.60220	0.0510*	
H6B	0.57530	1.08180	0.61070	0.0510*	

### Atomic displacement parameters (Å^2^)

**Table:**

	*U*^11^	*U*^22^	*U*^33^	*U*^12^	*U*^13^	*U*^23^
S1	0.0285 (2)	0.0340 (2)	0.0570 (2)	−0.0021 (1)	0.0095 (1)	−0.0001 (2)
O2	0.0284 (5)	0.0346 (5)	0.0788 (8)	−0.0014 (4)	0.0105 (5)	0.0016 (5)
O3	0.0434 (6)	0.0332 (6)	0.0928 (10)	0.0018 (5)	0.0154 (6)	0.0087 (6)
O4	0.0459 (6)	0.0783 (9)	0.0554 (7)	−0.0116 (6)	0.0117 (5)	−0.0091 (6)
C7	0.0265 (6)	0.0356 (7)	0.0519 (8)	−0.0021 (5)	0.0053 (5)	0.0004 (6)
C8	0.0409 (8)	0.0524 (10)	0.0600 (10)	−0.0048 (7)	0.0094 (7)	−0.0104 (8)
C9	0.0469 (9)	0.0889 (15)	0.0554 (10)	−0.0014 (10)	0.0140 (8)	−0.0057 (10)
C10	0.0336 (7)	0.0877 (15)	0.0618 (11)	−0.0043 (8)	0.0039 (7)	0.0239 (10)
C11	0.0458 (9)	0.0518 (11)	0.0791 (13)	−0.0138 (8)	−0.0004 (8)	0.0191 (9)
C12	0.0411 (7)	0.0379 (8)	0.0638 (10)	−0.0056 (6)	0.0047 (7)	−0.0005 (7)
C13	0.0533 (11)	0.149 (3)	0.0824 (15)	−0.0176 (14)	0.0114 (10)	0.0509 (16)
O1	0.0415 (5)	0.0209 (5)	0.0767 (8)	0.0010 (4)	0.0104 (5)	0.0001 (5)
N1	0.0338 (5)	0.0190 (5)	0.0395 (6)	−0.0006 (4)	0.0066 (4)	0.0008 (4)
N2	0.0333 (5)	0.0271 (6)	0.0552 (7)	0.0018 (4)	0.0092 (5)	0.0027 (5)
N3	0.0307 (5)	0.0221 (5)	0.0417 (6)	−0.0022 (4)	0.0061 (4)	0.0018 (4)
N6	0.0353 (6)	0.0269 (6)	0.0665 (8)	−0.0041 (5)	0.0126 (6)	−0.0005 (5)
C2	0.0332 (6)	0.0244 (6)	0.0293 (6)	−0.0003 (5)	0.0042 (4)	0.0014 (4)
C4	0.0346 (6)	0.0230 (6)	0.0388 (6)	0.0009 (5)	0.0040 (5)	0.0010 (5)
C5	0.0316 (6)	0.0256 (6)	0.0462 (7)	0.0008 (5)	0.0068 (5)	0.0001 (5)
C6	0.0328 (6)	0.0261 (6)	0.0338 (6)	−0.0020 (5)	0.0055 (5)	0.0008 (5)

### Geometric parameters (Å, º)

**Table:**

S1—O2	1.4728 (10)	C7—C12	1.383 (2)
S1—O3	1.4462 (12)	C7—C8	1.381 (2)
S1—O4	1.4444 (14)	C8—C9	1.389 (3)
S1—C7	1.7628 (15)	C9—C10	1.382 (4)
O1—C4	1.2416 (16)	C10—C13	1.511 (3)
N1—C6	1.3835 (16)	C10—C11	1.376 (3)
N1—C2	1.3442 (16)	C11—C12	1.388 (3)
N2—C2	1.3077 (18)	C8—H8	0.9300
N3—C2	1.3410 (16)	C9—H9	0.9300
N3—C4	1.3994 (16)	C11—H11	0.9300
N6—C6	1.3228 (18)	C12—H12	0.9300
N1—H1	0.8600	C13—H13C	0.9600
N2—H2A	0.8600	C13—H13A	0.9600
N2—H2B	0.8600	C13—H13B	0.9600
N3—H3	0.8600	C4—C5	1.3932 (18)
N6—H6B	0.8600	C5—C6	1.3725 (19)
N6—H6A	0.8600	C5—H5	0.9300
			
S1···H2A	3.0800	C5···O4^ii^	3.4031 (18)
S1···H2B^i^	3.0100	C6···N2^v^	3.2886 (19)
S1···H3	2.8600	C6···O1^iv^	3.1973 (16)
O1···C6^i^	3.1973 (16)	C6···C2^v^	3.5776 (18)
O1···N1^i^	2.6515 (14)	C13···O4^vi^	3.298 (3)
O1···N6^i^	2.8201 (15)	C4···H1^i^	3.0400
O1···C2^ii^	3.1894 (18)	C4···H6B^i^	3.0600
O2···N3	2.7689 (14)	C7···H13A^vii^	3.1000
O2···N2^i^	2.8669 (16)	C12···H13B^iii^	3.0100
O3···N2	2.7935 (17)	H1···H2B	2.3000
O4···C5^ii^	3.4031 (18)	H1···H6B	2.2200
O4···N6^ii^	2.9498 (18)	H1···C4^iv^	3.0400
O4···C13^iii^	3.298 (3)	H1···O1^iv^	1.8600
O1···H6B^i^	2.0800	H2A···H3	2.3000
O1···H1^i^	1.8600	H2A···S1	3.0800
O2···H3	1.9200	H2A···O3	1.9500
O2···H2B^i^	2.0100	H2B···H1	2.3000
O3···H12^iv^	2.8700	H2B···S1^iv^	3.0100
O3···H3	2.8400	H2B···O2^iv^	2.0100
O3···H8	2.6000	H3···S1	2.8600
O3···H2A	1.9500	H3···H2A	2.3000
O4···H5^ii^	2.8000	H3···O2	1.9200
O4···H13C^iii^	2.9100	H3···O3	2.8400
O4···H6A^ii^	2.2500	H5···O4^ii^	2.8000
O4···H12	2.6800	H5···H8^v^	2.5100
N1···O1^iv^	2.6515 (14)	H5···H6A	2.4600
N1···C2^v^	3.3319 (16)	H6A···O4^ii^	2.2500
N2···O3	2.7935 (17)	H6A···H5	2.4600
N2···O2^iv^	2.8669 (16)	H6B···C4^iv^	3.0600
N2···C6^v^	3.2886 (19)	H6B···H1	2.2200
N3···C5^ii^	3.4276 (18)	H6B···O1^iv^	2.0800
N3···O2	2.7689 (14)	H8···H5^v^	2.5100
N3···N3^v^	3.2844 (17)	H8···O3	2.6000
N3···C4^v^	3.4212 (18)	H9···H13B	2.4700
N3···C4^ii^	3.2207 (18)	H11···H13C	2.4100
N6···O1^iv^	2.8201 (15)	H12···O4	2.6800
N6···O4^ii^	2.9498 (18)	H12···H13B^iii^	2.5300
C2···N1^v^	3.3319 (16)	H12···O3^i^	2.8700
C2···C2^v^	3.3868 (17)	H13A···C7^viii^	3.1000
C2···C6^v^	3.5776 (18)	H13B···H9	2.4700
C2···O1^ii^	3.1894 (18)	H13B···C12^vi^	3.0100
C4···N3^ii^	3.2207 (18)	H13B···H12^vi^	2.5300
C4···C4^ii^	3.2446 (18)	H13C···H11	2.4100
C4···N3^v^	3.4212 (18)	H13C···O4^vi^	2.9100
C5···N3^ii^	3.4276 (18)		
			
O2—S1—O3	110.84 (7)	C7—C12—C11	119.19 (17)
O2—S1—O4	111.93 (7)	C9—C8—H8	120.00
O2—S1—C7	105.89 (7)	C7—C8—H8	120.00
O3—S1—O4	113.91 (8)	C10—C9—H9	119.00
O3—S1—C7	106.83 (7)	C8—C9—H9	119.00
O4—S1—C7	106.91 (7)	C10—C11—H11	119.00
C2—N1—C6	122.36 (11)	C12—C11—H11	119.00
C2—N3—C4	123.20 (10)	C11—C12—H12	120.00
C6—N1—H1	119.00	C7—C12—H12	120.00
C2—N1—H1	119.00	C10—C13—H13C	109.00
C2—N2—H2B	120.00	H13B—C13—H13C	109.00
H2A—N2—H2B	120.00	C10—C13—H13A	109.00
C2—N2—H2A	120.00	C10—C13—H13B	109.00
C4—N3—H3	118.00	H13A—C13—H13C	109.00
C2—N3—H3	118.00	H13A—C13—H13B	110.00
C6—N6—H6A	120.00	N1—C2—N3	118.20 (11)
H6A—N6—H6B	120.00	N1—C2—N2	120.67 (12)
C6—N6—H6B	120.00	N2—C2—N3	121.13 (11)
S1—C7—C12	119.60 (12)	N3—C4—C5	116.98 (11)
C8—C7—C12	119.99 (15)	O1—C4—C5	125.95 (12)
S1—C7—C8	120.41 (11)	O1—C4—N3	117.07 (11)
C7—C8—C9	119.68 (18)	C4—C5—C6	120.20 (12)
C8—C9—C10	121.11 (19)	N1—C6—N6	116.28 (12)
C11—C10—C13	120.3 (2)	N1—C6—C5	118.97 (11)
C9—C10—C13	121.5 (2)	N6—C6—C5	124.76 (12)
C9—C10—C11	118.19 (18)	C4—C5—H5	120.00
C10—C11—C12	121.79 (19)	C6—C5—H5	120.00
			
O2—S1—C7—C8	94.26 (13)	S1—C7—C12—C11	−179.44 (13)
O2—S1—C7—C12	−84.77 (13)	S1—C7—C8—C9	179.93 (13)
O3—S1—C7—C8	−23.93 (14)	C12—C7—C8—C9	−1.0 (2)
O3—S1—C7—C12	157.04 (12)	C8—C7—C12—C11	1.5 (2)
O4—S1—C7—C8	−146.24 (13)	C7—C8—C9—C10	−1.0 (3)
O4—S1—C7—C12	34.72 (14)	C8—C9—C10—C13	−176.68 (19)
C6—N1—C2—N2	−177.54 (12)	C8—C9—C10—C11	2.4 (3)
C6—N1—C2—N3	3.24 (18)	C13—C10—C11—C12	177.18 (19)
C2—N1—C6—N6	178.45 (12)	C9—C10—C11—C12	−2.0 (3)
C2—N1—C6—C5	−1.84 (19)	C10—C11—C12—C7	0.0 (3)
C2—N3—C4—O1	−176.29 (13)	O1—C4—C5—C6	177.72 (14)
C2—N3—C4—C5	2.62 (19)	N3—C4—C5—C6	−1.1 (2)
C4—N3—C2—N2	177.12 (12)	C4—C5—C6—N6	−179.57 (14)
C4—N3—C2—N1	−3.67 (18)	C4—C5—C6—N1	0.8 (2)

Symmetry codes: (i) *x*, *y*−1, *z*; (ii) −*x*+1, −*y*+1, −*z*+1; (iii) *x*, −*y*, *z*−1/2; (iv) *x*, *y*+1, *z*; (v) −*x*+1, *y*, −*z*+3/2; (vi) *x*, −*y*, *z*+1/2; (vii) −*x*+1/2, *y*+1/2, −*z*+3/2; (viii) −*x*+1/2, *y*−1/2, −*z*+3/2.

### Hydrogen-bond geometry (Å, º)

**Table:**

*D*—H···*A*	*D*—H	H···*A*	*D*···*A*	*D*—H···*A*
N1—H1···O1^iv^	0.86	1.86	2.6515 (14)	152
N2—H2*A*···O3	0.86	1.95	2.7935 (17)	166
N2—H2*B*···O2^iv^	0.86	2.01	2.8669 (16)	175
N3—H3···O2	0.86	1.92	2.7689 (14)	169
N6—H6*A*···O4^ii^	0.86	2.25	2.9498 (18)	139
N6—H6*B*···O1^iv^	0.86	2.08	2.8201 (15)	143

Symmetry codes: (ii) −*x*+1, −*y*+1, −*z*+1; (iv) *x*, *y*+1, *z*.
